# PRMEFNet: A Real-Time Unsupervised Multi-Exposure Fusion Network Driven by Prior Knowledge

**DOI:** 10.3390/jimaging12080389

**Published:** 2026-08-19

**Authors:** Junwei Qi, Hangdong Wang, Xu Xiao, Jingpeng Gao

**Affiliations:** 1 College of Information and Communication Engineering, Harbin Engineering University, Harbin 150001, China; qijunwei@hrbeu.edu.cn (J.Q.); whd@hrbeu.edu.cn (H.W.); xiaoxu@hrbeu.edu.cn (X.X.); 2 Key Laboratory of Advanced Marine Communication and Information Technology, Ministry of Industry and Information, Harbin 150001, China

**Keywords:** multi-exposure image fusion, prior knowledge guidance, unsupervised learning, image decomposition, one-dimensional lookup table

## Abstract

Due to the limited dynamic range of imaging sensors, most cameras can only capture low-dynamic-range (LDR) images. Multi-exposure fusion (MEF) is an effective technique for generating high-dynamic-range (HDR) images. However, to simultaneously preserve texture details and global exposure, most existing methods primarily rely on more complex models to improve performance, resulting in higher computational costs and longer processing times. To address this issue, we propose a real-time unsupervised MEF network driven by prior knowledge. To this end, a hierarchical feature extraction module is designed that utilizes filtering operations to decompose the source images into base layers and detail layers. Features are extracted from each layer separately to reduce the difficulty of extracting effective features. Then, the receptive field of feature maps is expanded by dilated convolutions, and a window-based self-attention mechanism is applied to perform context modeling, achieving effective contextual modeling with low computational cost. Subsequently, texture features and global features are extracted separately to enable the model to maintain both local texture clarity and global smoothness. In addition, a one-dimensional lookup table is utilized to accelerate the inference process. Comprehensive experiments are conducted to verify the effectiveness of the proposed method. The subjective evaluation results demonstrate that the fused images exhibit superior visual quality, while objective experiments further quantify its superior performance, demonstrating that the proposed method effectively reduces computation time.

## 1. Introduction

Due to the limited bit depth of imaging sensors, consumer digital cameras can only capture low-dynamic-range (LDR) images, which cannot fully represent the content of real scenes [1]. A prevalent solution is multi-exposure fusion (MEF), which integrates a sequence of LDR images with disparate exposure levels to yield a high-dynamic-range (HDR) image [2].

**Figure 1:**
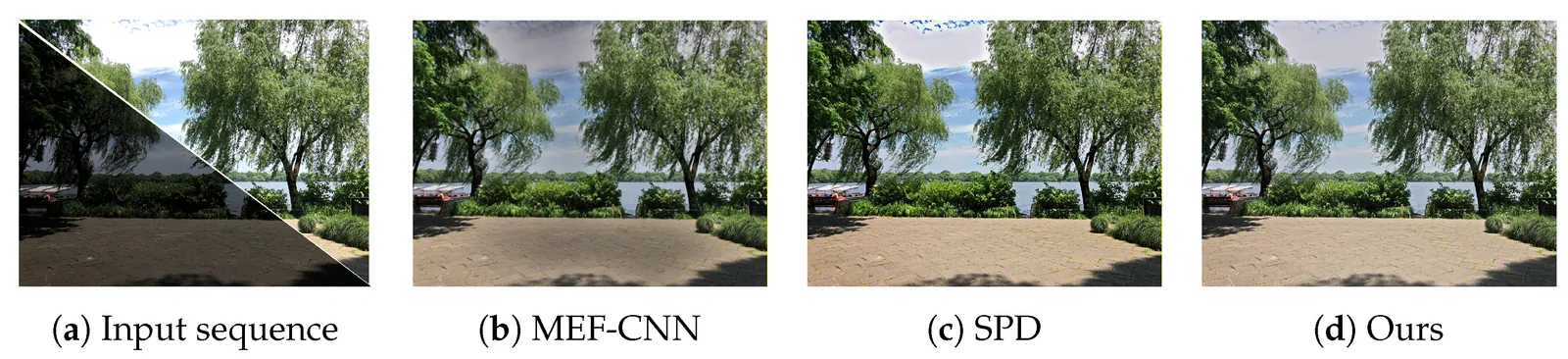
Visual comparison of different methods.

Traditional methods typically rely on fusion strategies that are manually designed based on the statistical characteristics of images [3,4,5]. However, such statistical features are not universally applicable to all scenarios, which limits their feature representation capability. Moreover, they primarily focus on low-level texture-feature extraction, neglecting the importance of high-level semantic information in MEF. As a result, the fused images often exhibit unnatural transitions and halo artifacts, as illustrated in Figure 1, where the SPD method [6] produces unnatural transitions in the cloud regions. With the advancement of deep learning for image-related tasks, deep learning-based MEF methods have demonstrated continually improving performance. However, most deep learning-based MEF methods neglect the use of prior knowledge and focus solely on increasing network complexity to improve fusion performance. Specifically, some methods focus on high-level semantic features [7,8], causing the network to bias toward global content understanding and resulting in suboptimal performance in texture detail preservation; while other methods [9,10] employ more complex network architectures in an attempt to simultaneously enhance global naturalness and detail retention. Although convolutional neural networks (CNNs) exhibit high inference speed, their performance degrades in tasks that require contextual information, such as luminance equalization and halo artifact suppression [11]; as shown in Figure 1, the MEF-CNN method produces unnatural dark regions in the sky. In contrast, Transformer-based architectures with self-attention mechanisms [12] can effectively capture long-range dependencies and improve fusion quality. However, their computational complexity grows sharply with increasing input-image resolution, leading to high inference costs [9]; this makes balancing performance and efficiency difficult. Furthermore, many existing methods rely on ground truth (GT) images for supervised training [13]. However, the GT images in mainstream MEF datasets (e.g., SICE) [14] are generated by existing methods, meaning that they serve only as pseudo-references rather than a true GT; this limitation restricts the upper performance bound of supervised methods. Supervised training may therefore inherit the luminance preferences, color styles, and potential artifacts of these pseudo-references. In contrast, the proposed unsupervised strategy directly utilizes complementary structural, contrast, and texture information from real multi-exposure images without relying on manually constructed fusion labels. This enables the model to learn complementary relationships among images with different exposure times, rather than simply reproducing the processing style of a specific pseudo-reference, and facilitates extending the training data to more real-world scenes.

To address the aforementioned issues, in this study the intrinsic characteristics of MEF are investigated. By analyzing the causes of halo artifacts and the reasons for the conflict between texture clarity and global consistency, we explore how prior knowledge is utilized to guide neural networks to perform more effective feature extraction and fusion according to the requirements of the MEF task. Based on these analyses, an unsupervised MEF network driven by prior knowledge is proposed. Prior knowledge generally refers to information that is available before model training and that originates from a source independent of the current training samples; it can be explicitly represented and integrated into the learning pipeline [15]. It may originate from physical mechanisms, domain principles, or stable signal characteristics and can be incorporated through model architectures or learning constraints [16]. In this study, prior knowledge specifically denotes stable characteristics inherent to multi-exposure images that are known before training. Based on this understanding, the task-specific prior knowledge of MEF is translated into structural constraints and embedded into different functional modules of the network, guiding the model to organize and represent information in a manner consistent with its distinct characteristics. This design retains the adaptability of data-driven learning while providing prior guidance for feature extraction and collaborative fusion, thereby guiding the model to extract features more effectively. Specifically, the network is composed of a detail feature extraction branch (DFEB) and a global feature extraction branch (GFEB). Each branch operates under distinct structural constraints and is respectively guided to capture texture and global features. The DFEB consists of the hierarchical feature extraction module (HFEM) and the local-context feature fusion module (LCFM). Within the HFEM, the input images are decomposed into base layers and detail layers through filtering operations, where the feature extractors independently capture base and detail features. To fully exploit the information contained in the source images, residual connections are introduced between the input and output. Subsequently, the LCFM integrates dilated convolutions with varying dilation rates and a window-based self-attention mechanism to fuse local contextual features, thereby achieving efficient context modeling with lower computational cost. The GFEB adopts a U-Net-style [17] encoder–decoder structure. In the encoding stage, multi-scale convolutions and max pooling are employed to rapidly expand the receptive field to a global scope. In the decoding stage, spatial resolution is progressively restored through multi-scale convolutions and upsampling, while residual connections are incorporated to achieve a balance between shallow and deep features. After obtaining the texture features and the global features, they are jointly used to generate the fused image, preserving both texture details and overall naturalness.

In summary, the main contributions of this study are as follows:

1. An unsupervised MEF network driven by prior knowledge is proposed. By introducing structural constraints to incorporate prior knowledge, the proposed network can regard the image as different layers with more stable statistical characteristics.
2. An HFEM and LCFM are designed for texture restoration. The HFEM decouples the base and detail layers to extract more effective features for texture preservation. Subsequently, based on a windowed self-attention mechanism, the LCFM efficiently fuses features extracted by the HFEM while alleviating the conflict between computational overhead and contextual modeling.
3. A collaborative fusion strategy is proposed to address the challenge that existing networks face in balancing texture features and global features. Specifically, a U-Net-based GFEB is constructed, in which a designed multi-scale convolution module is employed to guide global feature extraction. By collaboratively leveraging global and texture features, the proposed network effectively balances texture preservation and overall naturalness.

## 2. Related Work

In this section, we provide an overview of classical MEF methods. Based on the underlying techniques, these can be broadly categorized into traditional [18,19,20,21] and deep learning-based [22,23,24]. Traditional methods depend on handcrafted feature extraction and fusion schemes, which perform MEF in either the spatial or transform domain. Conversely, deep learning-based methods exploit the strong nonlinear modeling and feature-learning abilities of neural networks to directly learn the transformation from LDR inputs to HDR outputs.

### 2.1. Traditional MEF Methods

Traditional MEF techniques can be broadly categorized into spatial-domain and transform-domain methods [25]. The former performs fusion operations directly in the pixel domain and can be further divided into pixel-based, block decomposition-based, and optimization-based methods, depending on the specific fusion strategy employed. Pixel-based methods achieve image fusion by constructing weight maps related to pixel information. For instance, Liu et al. [26] developed a fusion method based on dense scale-invariant feature transforms, which effectively suppresses dynamic artifacts in the resulting images. Likewise, Lee et al. [5] introduced a method employing dual weighting functions during fusion to make full use of pixel-level information. Block decomposition-based methods first divide the source images into blocks and then design fusion rules for the decomposed blocks. Goshtasby et al. [27] initially introduced the concept of block-based MEF, while Ma et al. [6] further improved this technique by dividing each image block into signal intensity, signal structure, and mean intensity components. Optimization-based methods obtain weight maps by solving optimization or energy functions. Song et al. [28] adopted a gradient-based method to maximize brightness contrast in the image, whereas Zhang et al. [29] derived the fused image by optimizing the structural similarity index.

Transform domain-based methods first project the source images into a specific transform space, in which the integration of features is conducted, and then reconstruct the final image through an inverse transformation. Common transform-domain methods include multi-scale, gradient-based, and sparse representation-based methods. Multi-scale methods achieve multi-level feature fusion through pyramid decomposition or filtering operations. Mertens et al. [30] proposed an image fusion method based on Laplacian and Gaussian pyramids, which avoids the need for camera response curve calibration and simplifies the HDR image synthesis process. Li et al. [31] employed guided filtering prior to the fusion process to separate the image into a base layer and a detail layer, enabling finer control of the fusion process. Gradient-based methods construct fusion rules based on image gradient information. Zhang [32] used a two-dimensional Gaussian filter to compute image gradients and designed fusion strategies based on gradient magnitudes to enhance structural consistency. Sparse representation-based methods perform feature fusion through dictionary learning and sparse coding. Wang et al. [33] constructed a K-SVD dictionary from image sequences, applied sparse representation using a sliding window, and fused the sparse coefficients in a weighted manner to generate high-quality HDR results.

### 2.2. Deep Learning-Based MEF Methods

As deep learning has developed, it has demonstrated outstanding performance in numerous image processing tasks. Inspired by these achievements, many studies have applied deep learning-based methods to further improve the performance of MEF. According to their network architecture, deep learning-based MEF methods are typically divided into CNN-based and generative adversarial network (GAN)-based methods. The introduction of CNNs into MEF was first accomplished by Kalantari et al. [34], using convolutional networks to replace manually designed fusion strategies. Prabhakar et al. [35] developed an unsupervised deep learning method that employs a loss function derived from the structural similarity index, thereby eliminating the need for GT images. Han et al. [36] developed a deep perceptual enhancement network consisting of an image-detail extraction module and a color mapping module, capable of generating fused images with rich texture details. Liu et al. [37] incorporated a multi-scale attention mechanism into the HALDeR framework to expand attention coverage across multiple scales, thereby improving exposure correction accuracy and alleviating color distortion. Kirsten et al. [38] proposed MobileMEF, which employs efficient convolutional blocks and YUV-domain processing to support real-time MEF on hardware-constrained mobile devices. CNNs exhibit strong feature extraction capabilities, enabling automatic feature extraction and fusion, thereby replacing handcrafted fusion strategies. Nevertheless, because the receptive field of convolutional kernels is limited, CNNs have difficulty in modeling long-range dependencies and maintaining global consistency. To address this limitation, several recent fusion methods have been proposed that integrate CNN with Transformer [39,40]. For instance, TransMEF [9] presented a Transformer-based fusion model that employs a self-attention mechanism to model global dependencies across spatial regions, achieving enhanced fusion performance but at the cost of high computational complexity and sensitivity to input resolution. Ma et al. [41] introduced a general image fusion network built upon the Swin Transformer, which integrates an attention-guided cross-domain module to capture global representations. This network achieved outstanding results across various image fusion tasks; however, it was not specifically optimized for MEF applications.

GAN-based MEF methods model exposure variations as probabilistic distributions and generate HDR images through adversarial learning. Xu et al. [42] were the first to introduce generative adversarial networks into MEF. Le et al. [43] introduced a GAN framework based on continual learning, in which a memory-augmented model was trained to preserve knowledge acquired from previous tasks, effectively mitigating the problem of catastrophic forgetting during model training. Zhou et al. [44] proposed GIDGAN, which constructs dual discriminators using gradient and intensity information to jointly capture salient and geometric structures. This method effectively alleviates the mode-collapse problem in GAN and significantly improves the robustness of the fusion process.

## 3. Proposed Method

In this section, we begin by outlining the overall network architecture in Section 3.1. Then, Section 3.2 and Section 3.3 provide detailed descriptions of the HFEM and LCFM modules within the DFEB, respectively. Section 3.4 presents the architecture and realization of the GFEB. The loss functions adopted in our network are discussed in Section 3.5, and finally, Section 3.6 provides an explanation of how the network inference process is accelerated using a one-dimensional lookup Table (1D-LUT).

### 3.1. Network Architecture

**Figure 2:**
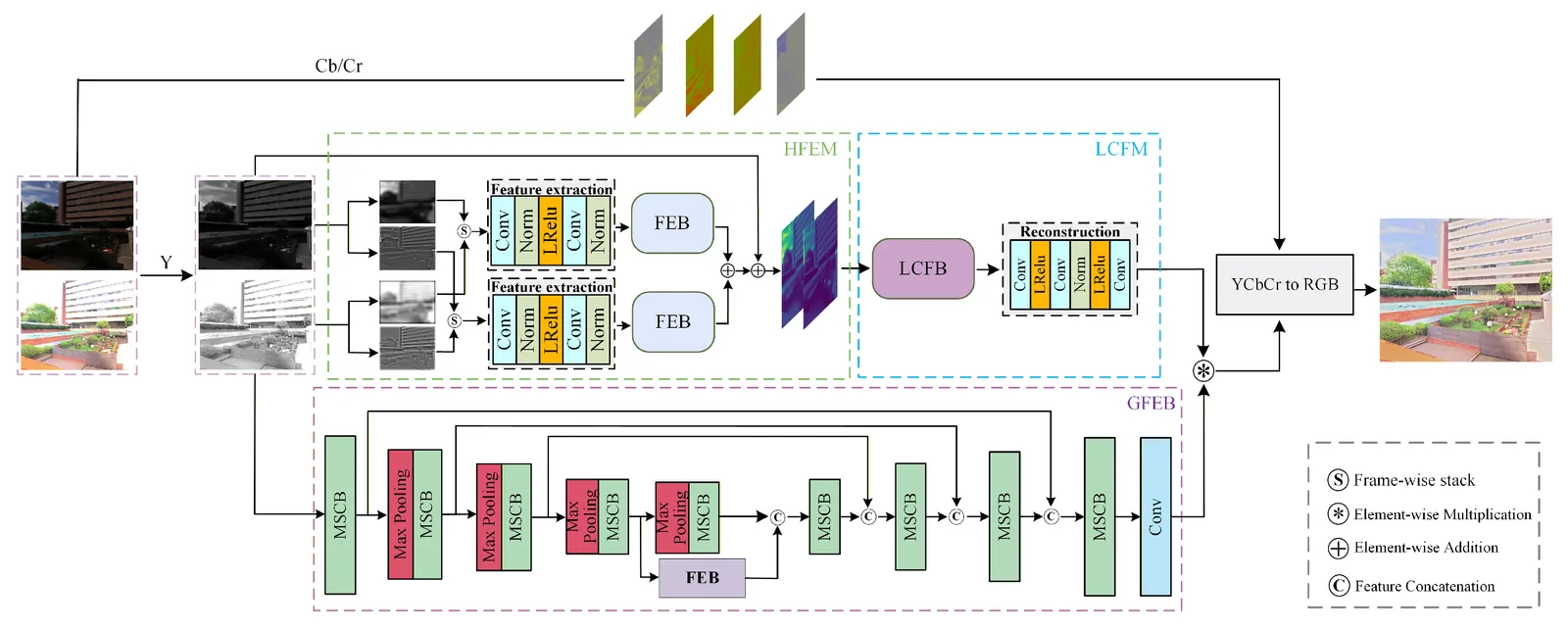
Overall network architecture.

The core idea of designing this network is to explicitly incorporate prior knowledge of the MEF task into the network architecture, thereby constructing a network that better fits the characteristics of this task. The network consists of two cooperative branches: a DFEB and a GFEB. The DFEB contains an HFEM and an LCFM. The HFEM decomposes the input images into base layers and detail layers, both exhibiting more stable statistical characteristics, which reduces the difficulty of feature extraction in complex scenes and enables the network to extract effective features more efficiently. The LCFM combines the Inception architecture [45] with dilated convolutions [46] to initially expand the receptive field of this branch. Subsequently, a window-based self-attention module is employed to strengthen the network’s capability in modeling local contexts without significantly increasing computational cost, thereby producing fused images with more natural texture transitions. This design primarily aims to enhance the network’s capacity for texture feature extraction. The GFEB, constructed on a U-Net architecture with custom-designed multi-scale convolutions, rapidly expands the receptive field to guide the network in capturing global semantic information, thereby reducing halo artifacts and improving the global naturalness of the fusion images. Finally, the fused image is produced through the integration of texture and global features. The overall network architecture is illustrated in Figure 2.

### 3.2. Hierarchical Feature Extraction Module

**Figure 3:**
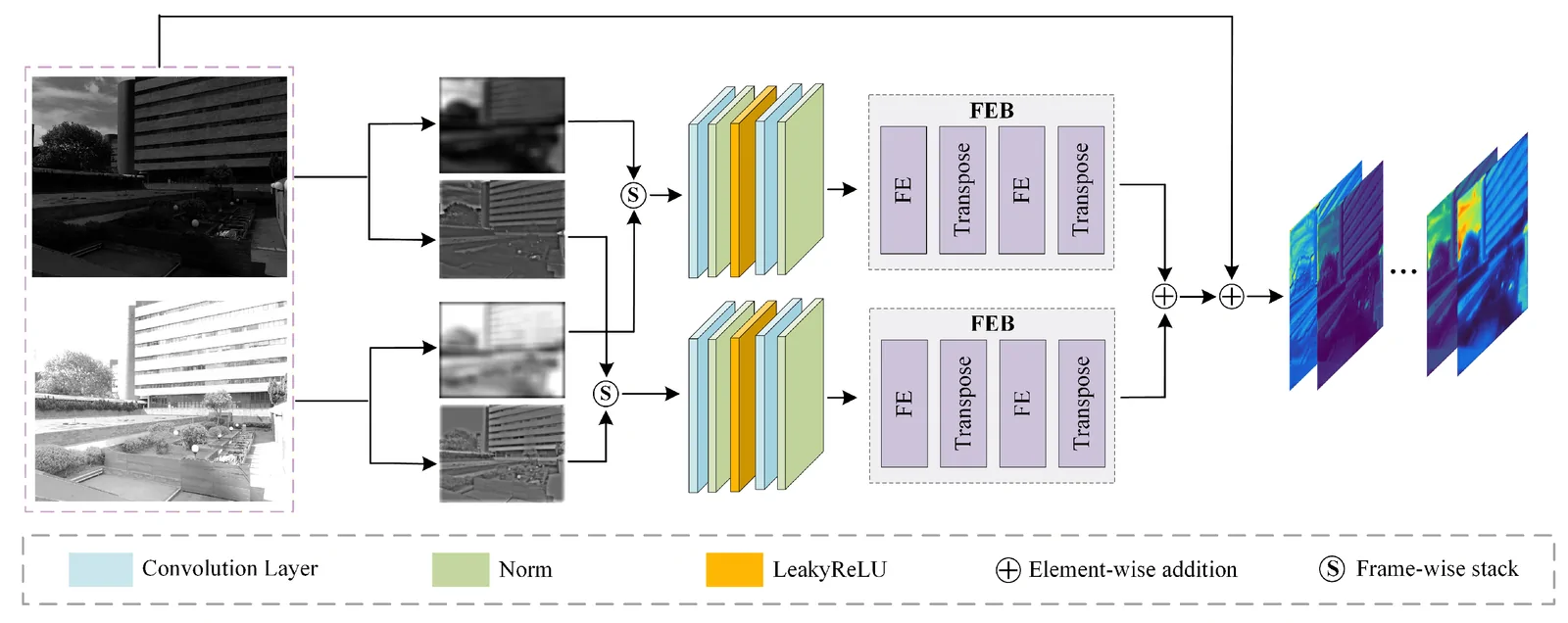
Network architecture of the HFEM.

The HFEM is designed to reduce the difficulty of extracting effective features directly from complex source images. In source images, global illumination variations and fine texture details are mixed together, making it difficult to directly design fusion rules. Traditional methods use image decomposition [31] to decouple these two types of features, allowing different fusion rules to be designed for different features. We incorporate this idea into our model design. The HFEM introduces image decomposition at the front end of the network and uses two feature extractors to extract the two types of features separately. Through this structural embedding, each feature path processes more concentrated and stable information, thereby reducing interference between different types of features and enabling the network to extract effective features more easily. The structure of the HFEM is illustrated in Figure 3.

**Figure 4:**
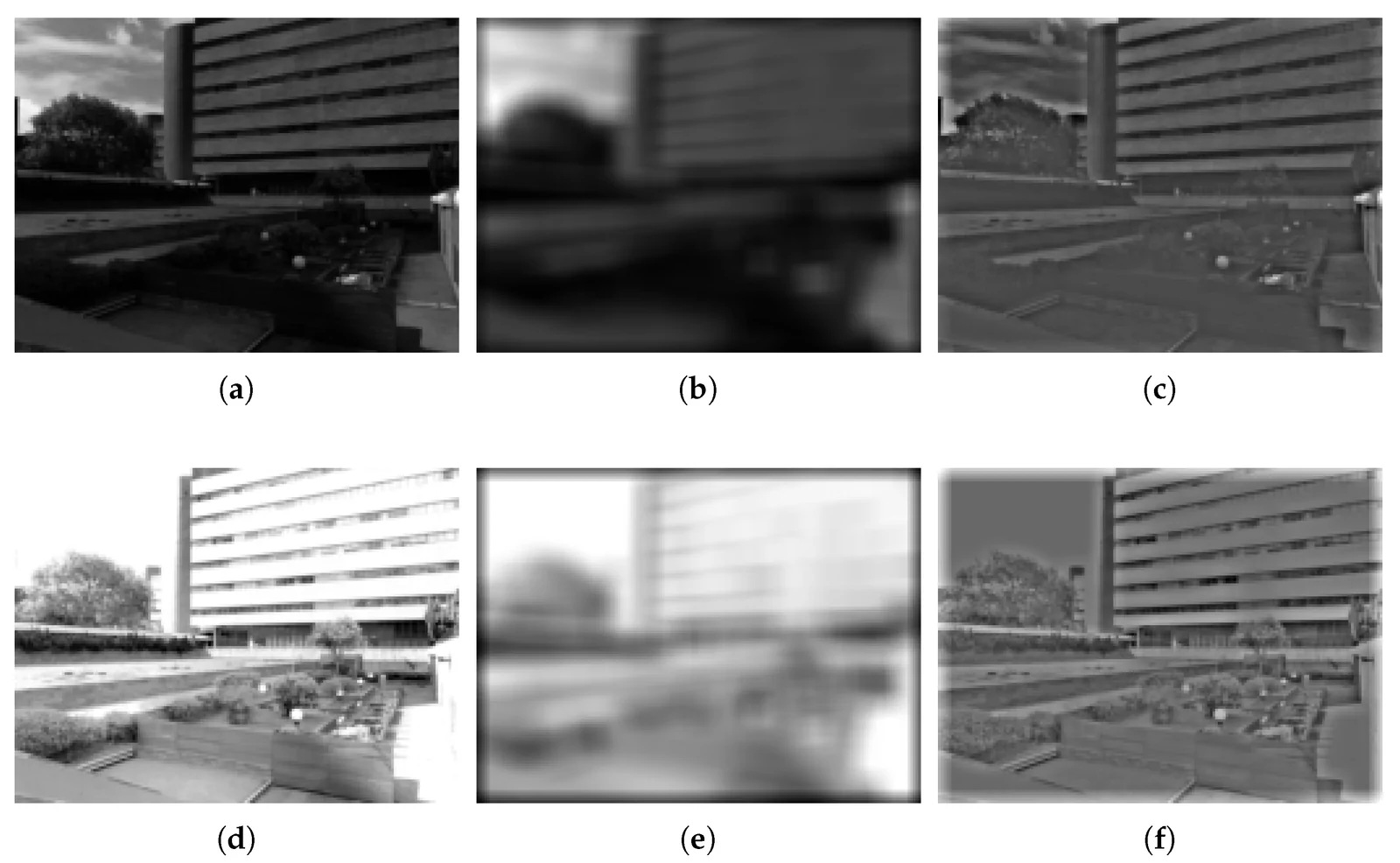
Visualization of image decomposition into base and detail layers: (**a**) under-exposed image; (**b**) base layer of the under-exposed image; (**c**) detail layer of the under-exposed image; (**d**) over-exposed image; (**e**) base layer of the over-exposed image; (**f**) detail layer of the over-exposed image.

A layering operation is first applied to the over-exposed and under-exposed images, $I_{u}$ and $I_{o}$, to provide structural priors for the HFEM; it can be formulated as follows:

(1) $$I_{o}^{\mathrm{base}}=\mathrm{MF}\left(I_{o},r\right),$$

(2) $$I_{u}^{\mathrm{base}}=\mathrm{MF}\left(I_{u},r\right),$$

(3) $$I_{o}^{\mathrm{detail}}=I_{o}-I_{o}^{\mathrm{base}},$$

(4) $$I_{u}^{\mathrm{detail}}=I_{u}-I_{u}^{\mathrm{base}},$$

where $I_{o}^{\mathrm{base}}$ and $I_{u}^{\mathrm{base}}$ represent the base layers of the over-exposed and under-exposed images, respectively, and $I_{o}^{\mathrm{detail}}$ and $I_{u}^{\mathrm{detail}}$ represent their corresponding detail layers; the function MF(·) denotes the mean filtering operation, and *r* represents the radius of the filter kernel. Through the above layering operation, the input images are decomposed into base and detail layers. Figure 4 illustrates the results of the image layering operation.

Subsequently, the base and detail layers of the source image sequence are stacked separately along the frame dimension as follows:

(5) $$F_{B}=\mathrm{Stack}\left(I_{o}^{\mathrm{base}},I_{u}^{\mathrm{base}}\right),$$

(6) $$F_{D}=\mathrm{Stack}\left(I_{o}^{\mathrm{detail}},I_{u}^{\mathrm{detail}}\right).$$

The stacked base and detail layers are then fed into the feature extractor $H_{\mathrm{FE}}$, yielding

(7) $$M_{B}=H_{\mathrm{FE}}\left(F_{B}\right),$$

(8) $$M_{D}=H_{\mathrm{FE}}\left(F_{D}\right),$$

where the feature extractor $H_{\mathrm{FE}}$ is composed of two 3×3 convolutional layers, two normalization layers, and an LReLU activation function, forming a lightweight fully convolutional module for feature extraction.

To obtain more effective feature representations, a feature enhancement block (FEB) is designed to enhance the extracted feature maps, enabling the model to focus more on informative features while reducing the influence of redundant information during parameter optimization. This module consists of two feature enhancement operations and two dimensionality exchange operations. The feature enhancement process, using the feature map from the base layer $M_{B}$ as an example, is described in detail in Equations (9)–(12).

First, global average pooling is applied to the input feature map $M_{B}\in R^{N\times C\times H\times W}$ to obtain the intensity information of each channel, thereby reflecting the importance of different channels:

(9) $$w_{1}^{n,c}=\frac{1}{HW}\sum_{i=1}^{H}\sum_{j=1}^{W}M_{B}^{n,c}\left(i,j\right),$$

where $w_{1}^{n,c}$ denotes the global average response of the *c*-th channel in the *n*-th frame. Subsequently, the channel descriptor vector obtained from global pooling is fed into the channel attention module, which employs two convolutional layers and a nonlinear mapping to generate the channel attention weights:

(10) $$w_{2}^{n,c}=\mathrm{Sigmoid}\;\left(\mathrm{Conv}\;\left(\mathrm{LReLU}\;\left(\mathrm{Conv}\;\left(w_{1}^{n,c}\right)\right)\right)\right).$$

The resulting channel attention weights $w_{2}^{n,c}$ are applied to the original feature map in a channel-wise manner to enhance informative features while suppressing redundant information:

(11) $$M_{\mathrm{FE}}=w_{2}^{n,c}\;\cdot \;M_{B}.$$

To prevent the attention mechanism causing excessive feature suppression, a residual connection is introduced, where the weighted features are added to the original features:

(12) $$M=\mathrm{LReLU}\left(M_{B}+M_{\mathrm{FE}}\right).$$

Considering the content complementarity among different source images in MEF, inter-frame feature enhancement is employed to promote information fusion across images captured under different exposure conditions. Specifically, the first two dimensions of the enhanced feature map $M\in R^{N\times C\times H\times W}$ are transposed to obtain $M^{T}\in R^{C\times N\times H\times W}$, and feature enhancement is further performed along the frame dimension to generate the final feature map:

(13) $$M_{\mathrm{FEB}}={\left(\mathrm{FE}\left(M^{T}\right)\right)}^{\mathrm{transpose}(0,1)}.$$

The source images contain a large amount of directly usable texture information; skip connections are introduced to fully exploit this information, thereby reducing the difficulty of feature extraction and improving feature fidelity. The final output feature map of the HFEM is expressed as follows:

(14) $$M_{\mathrm{HFEM}}=\mathrm{FEB}\left(M_{B}\right)+\mathrm{FEB}\left(M_{D}\right)+I$$

This feature map integrates both the detail and base information from images under different exposure conditions in the source image sequence, providing rich and effective texture features for subsequent context modeling and fusion modules.

### 3.3. Local-Context Fusion Module

After the HFEM, the source image sequence is encoded into a set of feature maps that focus on both base and detail information. To generate the fusion result, these features need to be fused in an efficient manner.

**Figure 5:**
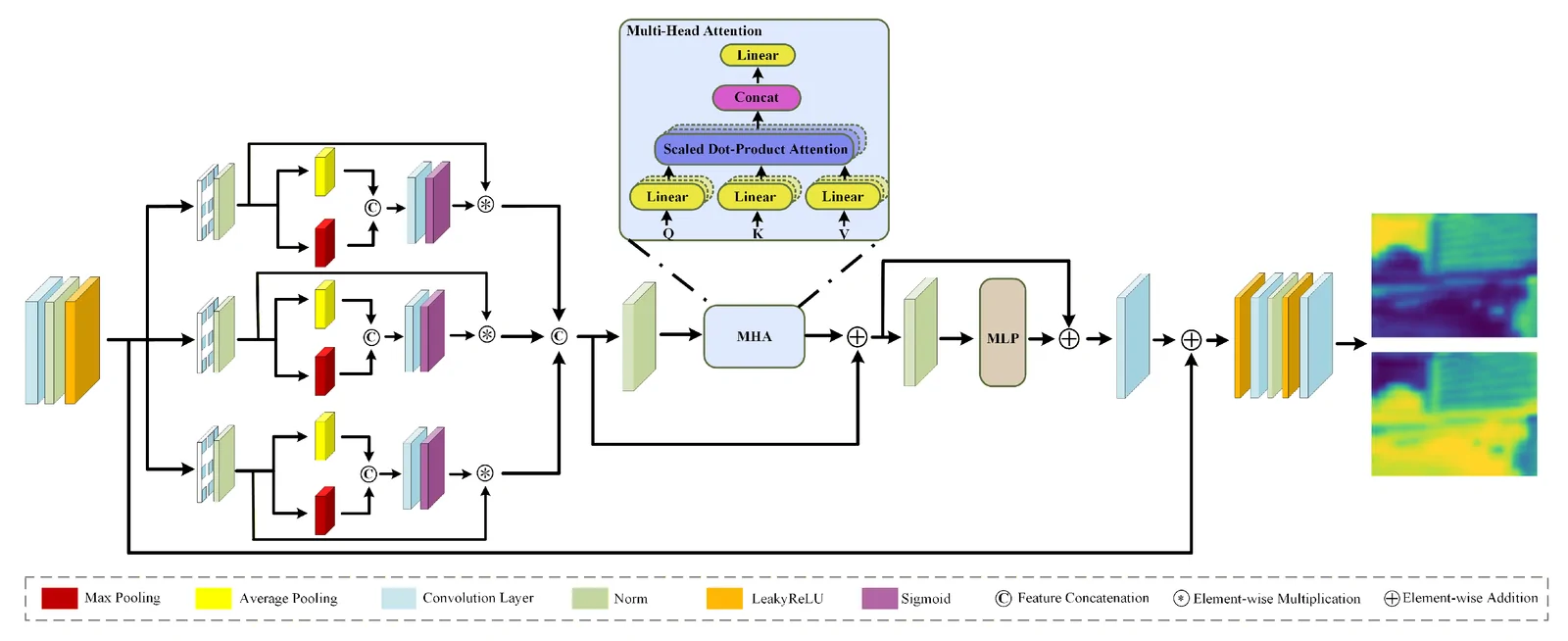
Network architecture of the LCFM.

The naturalness of texture transitions in MEF is primarily determined by the structural consistency within local neighborhoods. Therefore, an LCFM is designed in this study to achieve efficient feature fusion. The structure of the proposed module is illustrated in Figure 5.

The naturalness of texture transitions in fused images mainly depends on texture continuity within local neighborhoods. The LCFM embeds this prior knowledge into its multi-scale local feature extraction and contextual modeling structures. The module first employs an Inception structure combined with dilated convolutions for multi-scale feature extraction. Its parallel branches use convolutions with different dilation rates to capture neighborhood information at different scales, enlarging the receptive field without downsampling and thereby preserving texture details as much as possible. Subsequently, the LCFM employs window-based multi-head self-attention to establish content-dependent feature relationships within local windows and model the continuity between adjacent textures. This structure produces more natural texture transitions while avoiding the high computational cost of global self-attention, thereby balancing computational complexity and texture fusion quality.

The input feature map first passes through the input head:

(15) $$F_{0}=H\left(M_{\mathrm{HFEM}}\right),$$

where H(·) denotes a mapping function consisting of a convolutional layer, a normalization layer, and a nonlinear activation function LReLU, which is used to adjust the channel dimensions and normalize the distribution of the input features.

Three parallel convolutional branches are introduced to extract features from $F_{0}$, with each branch employing a different dilation rate to capture multi-scale contextual information:

(16) $$F_{2}=H_{2}\left(F_{0}\right),$$

(17) $$F_{4}=H_{4}\left(F_{0}\right),$$

(18) $$F_{8}=H_{8}\left(F_{0}\right),$$

where $H_{2}$, $H_{4}$, and $H_{8}$ represent dilated convolutional feature extractors with dilation rates of 2, 4, and 8, respectively. The extracted features are then weighted by the attention mechanism to highlight salient information, and the attention weights are computed as follows:

(19) $$W_{\mathrm{att}}=\mathrm{Sigmoid}\left(\mathrm{Conv}\left(\mathrm{Concat}\left(\mathrm{AvgPool}\left(F\right),\mathrm{MaxPool}\left(F\right)\right)\right)\right).$$

The outputs from each branch, after attention weighting, are concatenated to form an aggregated feature map, which is then further processed for context modeling:

(20) $$F_{\mathrm{cat}}=\mathrm{Concat}\left(W_{\mathrm{att}2}\;\cdot \;F_{2},W_{\mathrm{att}4}\;\cdot \;F_{4},W_{\mathrm{att}8}\;\cdot \;F_{8}\right),$$

where $W_{\mathrm{att}2}$, $W_{\mathrm{att}4}$, and $W_{\mathrm{att}8}$ are the attention weights computed using $F_{2}$, $F_{4}$, and $F_{8}$ as inputs.

Although dilated convolutions can expand the local receptive field to a certain extent, their ability to model complex contextual relationships remains limited. To further capture feature dependencies within local regions, a window-based multi-head self-attention module is introduced to enhance the model’s contextual modeling capability. Unlike traditional Transformers, which perform global self-attention over the entire feature map, this module computes self-attention weights independently within each local window. This design enables local-context modeling while significantly reducing computational complexity, thereby achieving natural transitions of texture details in the fusion result.

To verify the effectiveness of the DFEB, its feature maps are visualized in Figure 7. The feature maps from this branch form clear boundaries between different regions, indicating that the branch is capable of effectively extracting detail information.

### 3.4. Global Feature Extraction Branch

**Figure 6:**
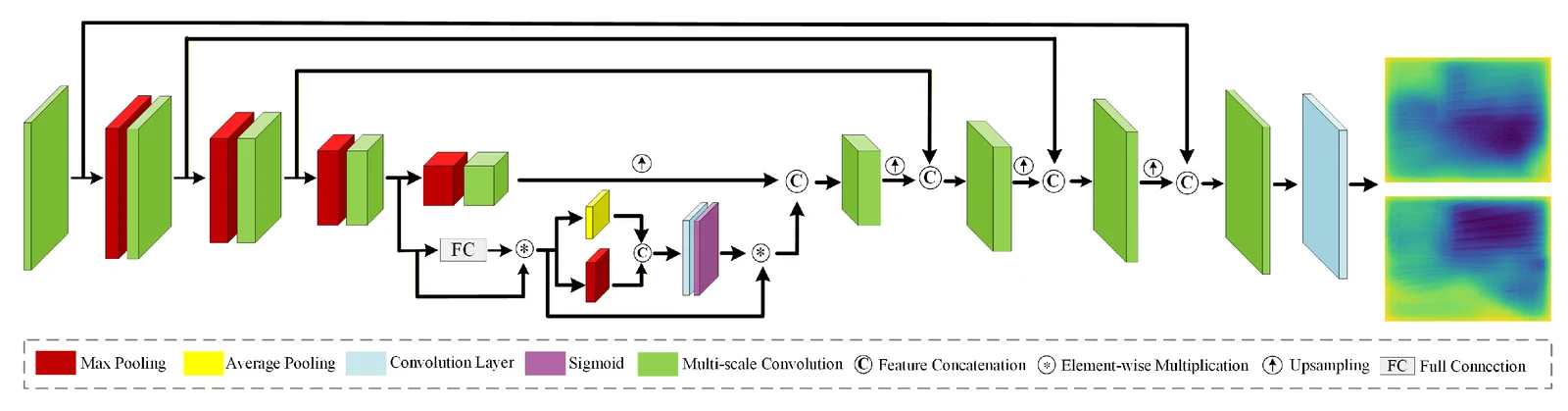
Network architecture of the GFEB.

In the DFEB, the model primarily focuses on detail extraction and fusion; however, excessive emphasis on detail results in insufficiently smooth transitions in the fusion weight map, leading to halo artifacts in regions with significant brightness variations. To alleviate this, a GFEB is introduced. Local texture features alone are insufficient to ensure the global naturalness of the fusion result and may produce halo artifacts around regions with large luminance differences. To avoid weakening texture details by simply deepening the network to enlarge the receptive field, this work adopts a dual-branch structure to extract local texture features and global abstract features separately. In the GFEB, the U-Net architecture and dilated convolutions are used to impose structural constraints, enabling this branch to rapidly enlarge its receptive field and extract global abstract features from the image. It thereby provides scene-level guidance for the locally focused DFEB and promotes smooth transitions between regions with different exposure levels. The structure of the GFEB is shown in Figure 6.

Specifically, this branch adopts a U-Net architecture with an encoder–decoder structure. In the encoding phase, the network enables the receptive field of the branch to rapidly expand to the global scale through multi-scale convolutions and max pooling. The multi-scale convolution module consists of three serially connected dilated convolution layers, which can be formulated as follows:

(21) $$F_{\mathrm{ms}}\left(X\right)={\mathrm{Conv}}_{\left(3,4\right)}\;\left({\mathrm{Conv}}_{\left(3,2\right)}\;\left({\mathrm{Conv}}_{\left(3,1\right)}\;\left(X\right)\right)\right),$$

where ${\mathrm{Conv}}_{\left(3,1\right)}$, ${\mathrm{Conv}}_{\left(3,2\right)}$, and ${\mathrm{Conv}}_{\left(3,4\right)}$ denote dilated convolution operations using a 3×3 kernel with dilation rates of 1, 2, and 4, each followed by normalization and LReLU activation functions. This design enables the GFEB to rapidly expand its receptive field to the global scale, guiding the branch to focus on global features.

In the decoding stage, the output of the decoder is progressively fused with the output of the encoder through skip connections. To enhance the robustness of the features, attention weighting is applied to the deepest skip connection before it is fused with the decoder features. Specifically, we first apply channel attention weighting, assigning adaptive weights to different feature maps along the channel dimension, which can be formulated as

(22) $$S_{c}\left(X\right)=X⊙\mathrm{Sigmoid}\left(\mathrm{Conv}\left(\mathrm{ReLU}\left(\mathrm{Conv}\left(\mathrm{Avg}(X)\right)\right)\right)\right),$$

where ⊙ denotes element-wise multiplication; Avg(·) represents the average computation over each channel; and Conv is a 1×1 convolution layer that facilitates information interaction and dimensional transformation between channels, effectively functioning as a fully connected layer. Subsequently, spatial attention weighting is applied, which can be expressed as

(23) $$S_{s}\left(X\right)=X⊙\mathrm{Sigmoid}\left(\mathrm{Conv}\left(\mathrm{Concat}\left(\mathrm{AvgPool}(X),\mathrm{MaxPool}(X)\right)\right)\right),$$

where AvgPool(·) and MaxPool(·) conduct average and max pooling on the feature map, respectively, to extract salient spatial representations, Concat(·) denotes the concatenation operation that merges the outputs from both pooling branches, and the convolution layer is utilized to produce the spatial attention weights. Finally, the feature map is enhanced by jointly applying the channel and spatial attention mechanisms:

(24) $$F_{\mathrm{att}}\left(X\right)=S_{s}\left(S_{c}\left(X\right)\right).$$

**Figure 7:**
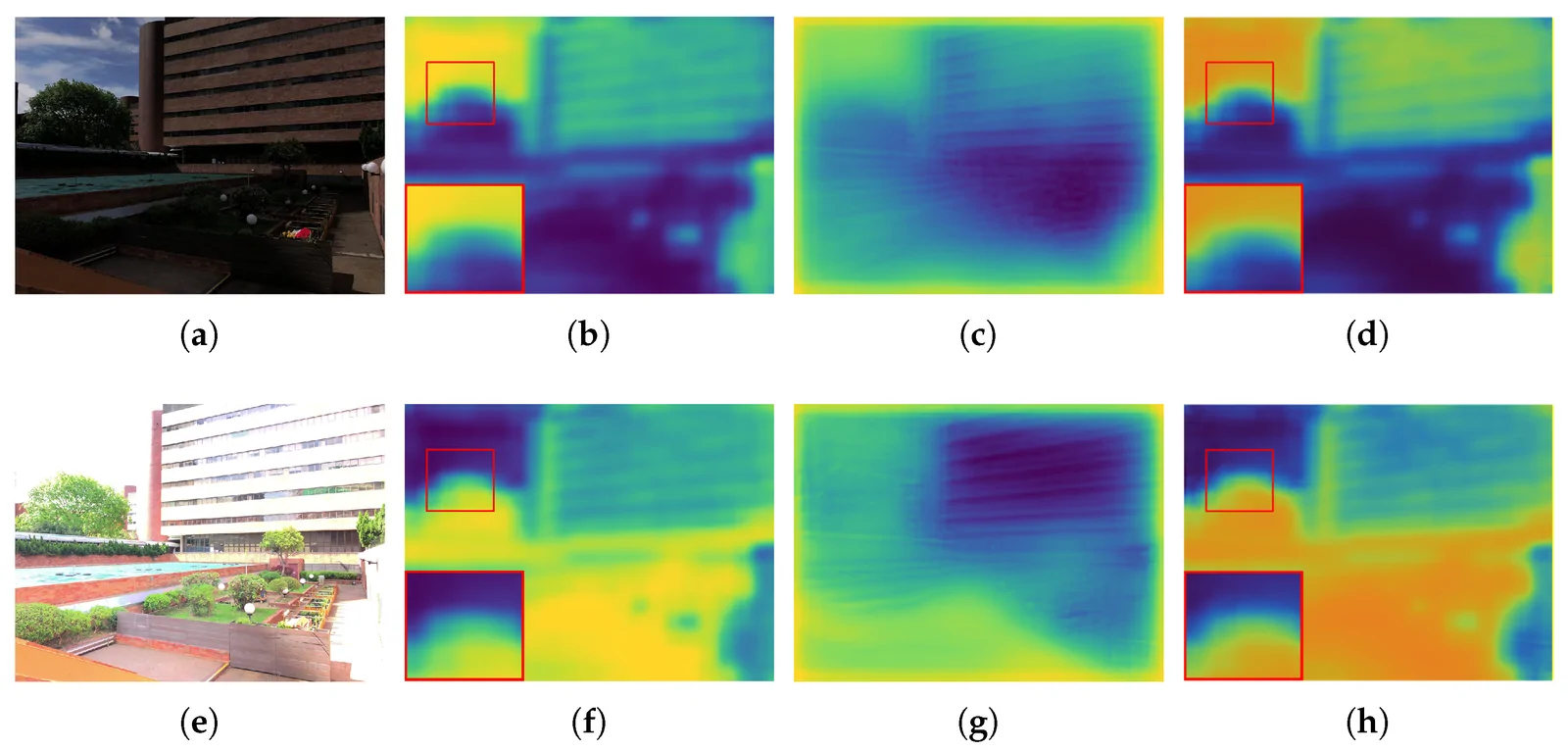
Visualization of feature maps from different branches: (**a**) under-exposed image; (**b**) DFEB feature map of the under-exposed image; (**c**) GFEB feature map of the under-exposed image; (**d**) final feature map of the under-exposed image; (**e**) over-exposed image; (**f**) DFEB feature map of the over-exposed image; (**g**) GFEB feature map of the over-exposed image; (**h**) final feature map of the over-exposed image.

As shown in Figure 7, the feature maps from the GFEB exhibit smoother transitions compared to those from the DFEB. Finally, the weight map generated by this branch is fused with that produced by the DFEB to form the final feature map, which effectively balances both detailed and global features. The fused feature map shows smoother transitions in regions with large exposure differences, effectively suppressing the occurrence of halo artifacts.

### 3.5. Loss Function

Due to their typically being a lack of reliable GT images in MEF, an unsupervised network is proposed in this study. In designing the loss function, the MEF-SSIM metric [47] with the luminance component removed is employed. The MEF-SSIM redefines three components of SSIM; it can be expressed as

(25) $$\mathrm{MEF}\text{-}\mathrm{SSIM}\left(F,G\right)=\frac{1}{W}\sum_{k=1}^{W}\left[\;l\left(f_{k},g_{k}\right)\;\right]\;\cdot \;\left[\;c\left(f_{k},g_{k}\right)\;\right]\;\cdot \;\left[\;s\left(f_{k},g_{k}\right)\;\right],$$

where *l*, *c*, and *s* represent the luminance, contrast, and structural components, respectively, $f_{k}$ and $g_{k}$ denote the *k*-th local patches of *F* and *G*, and *W* is the total number of sliding windows.

Considering that the proposed LCFM and GFEB possess global modeling capabilities and strong exposure recovery abilities, we exclude the luminance component from the designed loss function to allow the model to focus more on preserving texture details. However, removing the luminance component does not imply that exposure recovery is neglected. The GFEB leverages global information to provide overall exposure guidance for the fusion process, ensuring the naturalness of the fused image and smooth transitions between regions with different exposure levels. The loss function retains the contrast and structure terms to concentrate on texture and structure reconstruction. In the process of reconstructing textures, it drives the network to adaptively adjust the fusion weights of differently exposed images, thereby achieving luminance and exposure recovery. Consequently, the loss function is reformulated as follows:

(26) $$L_{\mathrm{MEF}\text{-}\mathrm{SSIM}}=-\frac{1}{W}\sum_{k=1}^{W}\left[\;c\left(f_{k},g_{k}\right)\;\right]\;\cdot \;\left[\;s\left(f_{k},g_{k}\right)\;\right].$$

This design relaxes the luminance constraint, enabling the network to focus more on restoring texture details during training. As a result, the model can generate fusion outputs with clearer textures and smoother transitions under unsupervised conditions.

### 3.6. Accelerating Inference Using 1D-LUT

To improve the fusion speed of the proposed network, 1D-LUT [48] is employed for inference acceleration. It should be emphasized that complete PRMEFNet inference performs the full feature-extraction and contextual-modeling processes. In contrast, the 1D-LUT is an approximation derived from the complete model. During 1D-LUT inference, only the mapping from pixel intensity to fusion weight is retained, while the complete spatial feature-extraction and contextual-modeling processes are no longer executed. Therefore, 1D-LUT inference is not equivalent to complete PRMEFNet inference.

First, the lookup table matrix is constructed as follows:

(27) $$L\in R^{K\times 256},$$

where *K* denotes the number of input source images in the sequence. The *k*-th row of the matrix corresponds to the 1D-LUT of the *k*-th source image. Let the trained multi-exposure fusion network be represented as H(·). To construct the 1D-LUT, 256 test samples with constant pixel intensities are sequentially input into the network, where the intensity value *v* gradually varies from 0 to 255. For the *v*-th input image, the pixel values of all source images are uniformly set to *v*, which can be expressed as

(28) $$X_{k}^{v}=v,\;k\in 1,2,\ldots ,K,\;v\in 0,1,\ldots ,255.$$

For each test sample, the network H(·) outputs the corresponding weight map ${\tilde{W}}_{k}^{v}$. The spatial average of each weight map is then calculated to obtain the weight:

(29) $$\omega_{k}^{v}=\frac{1}{HW}\sum_{i=1}^{H}\sum_{j=1}^{W}{\tilde{W}}_{k}^{v}\left(i,j\right).$$

Subsequently, the obtained scalar weights are stored in the lookup table matrix:

(30) $$L\left(k,v\right)=\omega_{k}^{v},\;k\in 1,2,\ldots ,K,\;v\in 0,1,\ldots ,255.$$

After all 256 sets of intensity inputs have been processed, the 1D-LUT matrix *L* is fully constructed.

In the inference phase, for any input pixel value p∈0,1,…,255, the corresponding fusion weight can be efficiently obtained from the lookup table. Before fusion, the corresponding LUT weights from different source images are normalized to obtain $\tilde{L}\left(k,p_{k}\left(i,j\right)\right)$. The fusion operation can be described as follows:

(31) $$Y\left(i,j\right)=\sum_{k=1}^{K}\tilde{L}\left(k,p_{k}\left(i,j\right)\right)\;\cdot \;p_{k}\left(i,j\right),$$

where $p_{k}\left(i,j\right)$ denotes the pixel intensity at position (i,j) in the *k*-th source image. By integrating the network with the 1D-LUT, high-quality fusion can be achieved efficiently without requiring model forward propagation.

## 4. Experiments

In this section, we present the results of a series of systematic experiments conducted to comprehensively evaluate the performance of the proposed method. First, we provide information about the experimental setup, including the datasets, training details, evaluation metrics, and comparison methods. Then, we describe both subjective and objective experiments, which were conducted with nine advanced methods to evaluate the effectiveness of our method, and the inference speeds are compared. Finally, the results of ablation studies are provided to demonstrate the roles of key design components in the model.

### 4.1. Dataset and Training Details

The experiments are conducted using the widely used SICE dataset [14]. A total of 489 image sequences are randomly selected for training and another 100 for validation, where each sequence contains one under-exposed image and one over-exposed image. Each two-exposure image sequence consists of the darkest and brightest images from the corresponding sequence in the SICE dataset. Additionally, we conduct cross-dataset experiments on the dataset released by Zhang et al. in PESPD [19] to further evaluate the generalizability of our method. During testing, all image sequences in the PESPD dataset were used. We selected the darkest and brightest images from each image sequence as the source images for model input.

During both the training and validation stages, the source images are first converted into the YCbCr color space to obtain their respective luminance and chrominance components, denoted as $I_{u}^{Y}$, $I_{u}^{Cb}$, $I_{u}^{Cr}$, $I_{o}^{Y}$, $I_{o}^{Cb}$, and $I_{o}^{Cr}$. Subsequently, the luminance components $I_{u}^{Y}$ and $I_{o}^{Y}$ from the under-exposed and over-exposed images are fed into the trained fusion network to generate the fused luminance component $I_{f}^{Y}$. The chrominance components are fused using a weighted summation method, as formulated below:

(32) $$\begin{array}{cc} \; & I_{f}^{Cb}=\frac{I_{u}^{Cb}\left(\right|I_{u}^{Cb}-\tau |)+I_{o}^{Cb}\left(\right|I_{o}^{Cb}-\tau \left|\right)}{|I_{u}^{Cb}-\tau |+|I_{o}^{Cb}-\tau |}, \end{array}$$

(33) $$\begin{array}{cc} \; & I_{f}^{Cr}=\frac{I_{u}^{Cr}\left(\right|I_{u}^{Cr}-\tau |)+I_{o}^{Cr}\left(\right|I_{o}^{Cr}-\tau \left|\right)}{|I_{u}^{Cr}-\tau |+|I_{o}^{Cr}-\tau |}. \end{array}$$

Finally, the fused luminance component $I_{f}^{Y}$ and the computed chrominance components $I_{f}^{Cb}$ and $I_{f}^{Cr}$ are jointly used to perform the inverse transformation from the YCbCr to the RGB color space, generating the final color-fused image.

The experiments are conducted on a computing platform equipped with an NVIDIA GeForce RTX 4090 GPU and an Intel Ultra 7 CPU, using Python 3.10, PyTorch 2.8, and CUDA 12.9. The random seed is fixed to 2025. The batch size is set to 4, the number of training epochs is 20, the Adam optimizer is adopted, and the learning rate is set to $1\times 10^{-4}$. The threshold τ is set to 128. The mean-filter radius used in the model is set to r=5. In the LCFM, the dilation rates of the three parallel dilated convolutional branches are set to 2, 4, and 8, respectively. Window-based multi-head self-attention uses an 8×8 local window with four attention heads.

### 4.2. Evaluation Metrics

The evaluation metric system adopted in this study employs traditional metrics based on images’ statistical properties together with advanced perceptual metrics derived from the human visual mechanism, providing a comprehensive evaluation from multiple dimensions.

Specifically, MEF-SSIM [47] measures the similarity between the fused image and the input-image sequence in terms of luminance, contrast, and structure through a structural similarity index, while $Q^{AB/F}$ [49] is a classical fusion-quality metric based on image gradients and contrast, used to evaluate the visual consistency of the fused image. These two metrics are the most commonly used in MEF evaluation. The entropy (EN) [50] reflects the amount of information contained in the fusion result, while the mutual information (MI) [47] represents the degree of information complementarity between the fused image and the source images; a higher MI value indicates that more source information is preserved during the fusion process. The standard deviation (SD) [51] characterizes the overall image contrast, whereas the spatial frequency (SF) [52] describes the frequency of spatial texture variations; both are used to evaluate the texture clarity of the fused image. In addition, the visual information fidelity (VIF) [53] and the average gradient (AG) [54] are utilized to assess the image performance in terms of visual fidelity and local gradient strength, reflecting the texture preservation ability of the fused image. The feature mutual information (FMI) [55] evaluates the degree of feature-level information sharing between the fused image and the source images, effectively reflecting the fidelity and detail preservation capability of the fusion process.

### 4.3. Comparative Methods

Comprehensive comparative experiments are conducted with nine advanced MEF methods: eight advanced deep learning-based methods, TransMEF [9], HALDeR [37], CFNet [56], MEFLUT [48], EAT [13], HoLoCo [57], GIFNet [10], and MobileMEF [38]; and one of the latest traditional methods, PESPD [19], which performs fusion based on image blocks. To ensure fairness in comparison, all fusion results are generated using the publicly released source codes and default parameter settings provided by the authors of each method. This setup guarantees the objectivity of the comparison.

### 4.4. Subjective Experiment

The visual quality of the proposed method and other comparison methods is evaluated on two representative sequences from the SICE dataset. Since the SICE dataset provides GT images, we not only present the fusion results of each method but also provide the corresponding signal intensity distribution maps compared with the GT, enabling a more intuitive comparison of fusion quality. From the overall visual perspective, the proposed method demonstrates superior fusion quality.

**Figure 8:**
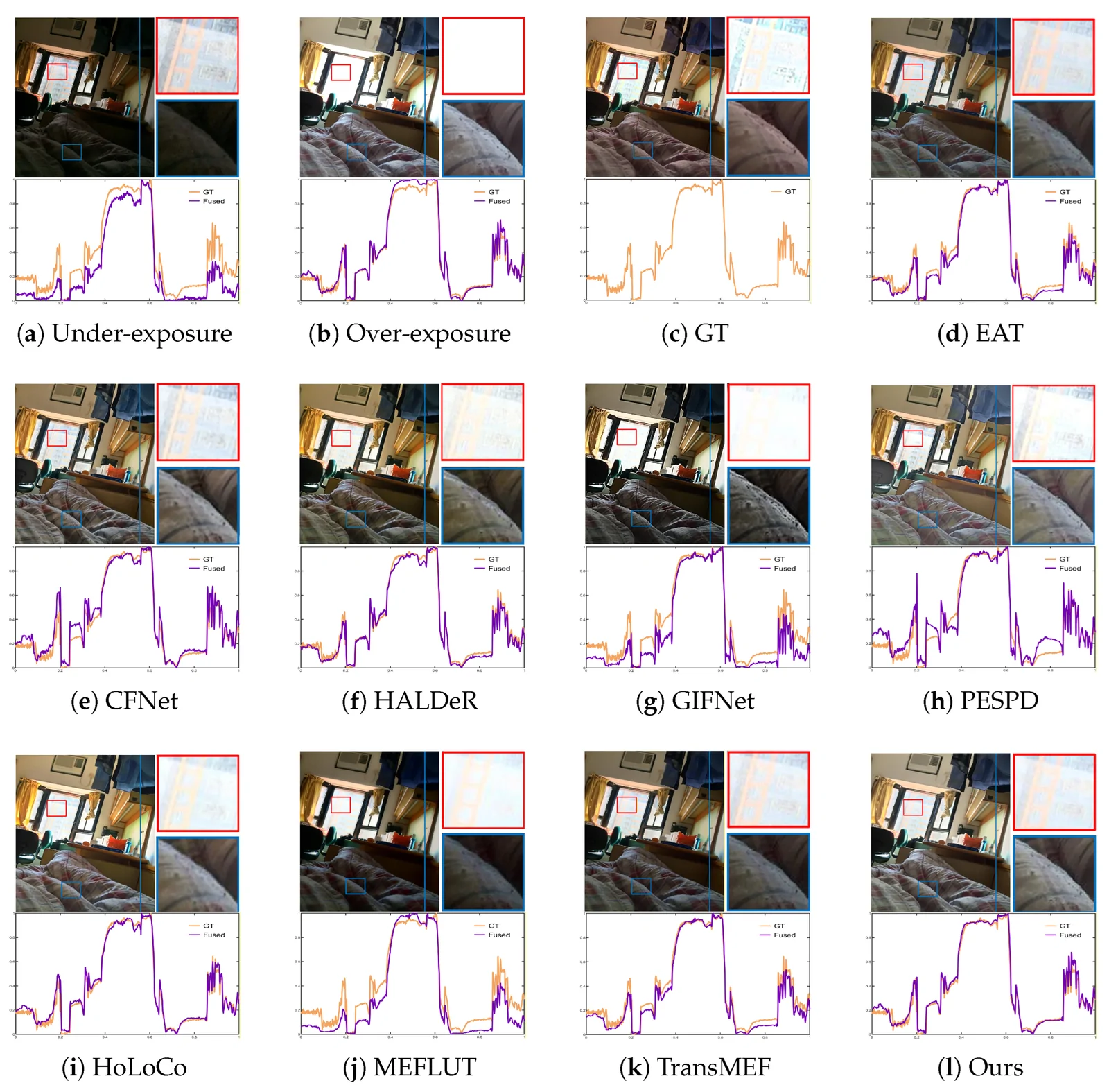
Visual comparison of different MEF methods on the “Room” scene. Each result shows the fused image and the corresponding pixel intensity comparison.

As illustrated in Figure 8, for the “Room” sequence, the images produced by TransMEF, MEFLUT, and GIFNet appear generally darker and fail to effectively recover the texture details in the inner region of the table. PESPD exhibits noticeable halo artifacts around the window area. The image generated by HALDeR loses part of the texture information in the blanket region, while HoLoCo preserves texture details but the overall image appears slightly blurred. In contrast, the proposed method achieves superior detail restoration in both the window and blanket regions, accurately reconstructing color and luminance information, producing a fused image that bears a closer resemblance to the GT image.

From the signal intensity distribution maps, it can be observed that the pixel intensities of TransMEF, MEFLUT, and GIFNet are generally lower than those of the GT image, whereas PESPD exhibits significantly higher pixel intensities in certain regions. Combined with the visual fusion results, these distribution differences are consistent with subjective perception: the former methods produce darker overall images, while the latter lose detail in the highlight areas. In contrast, the proposed method and HoLoCo exhibit signal distributions most consistent with the GT, indicating a more accurate luminance recovery capability.

**Figure 9:**
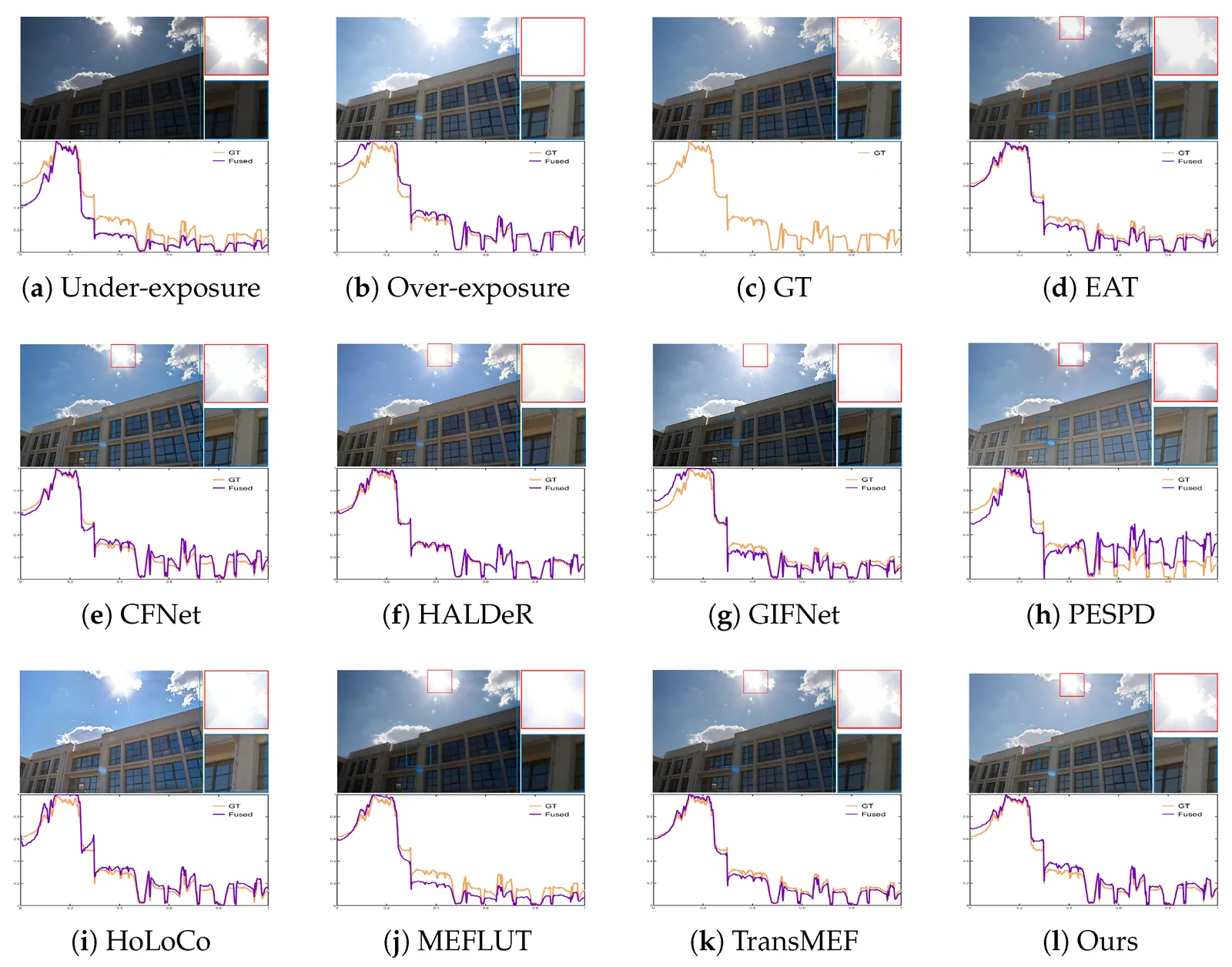
Visual comparison of different MEF methods on the “Outdoor” scene. Each result shows the fused image and the corresponding pixel intensity comparison.

To assess the robustness of the proposed method under extreme exposure conditions, a challenging scene with a large dynamic range was selected. The scene exhibits a wide range of brightness levels, and to preserve information in both bright and dark regions, the source image sequence was captured with significant exposure variations. As shown in Figure 9, the source image sequence exhibits severe under-exposure and over-exposure in the building surface and sun regions, respectively, resulting in a degradation of fusion performance across all methods.

Among the different methods, PESPD and HoLoCo preserve luminance and texture details well on the building surface, with HoLoCo also demonstrating excellent color recovery in the sky. However, both methods exhibit significant over-exposure in the sun area. In contrast, EAT and TransMEF perform better in the sun region but show insufficient overall brightness, causing loss of detail on the building surfaces due to under-exposure. GIFNet and MEFLUT fail to achieve reasonable exposure restoration in both the bright and dark regions. HALDeR performs well on the building surface, but its restoration of the sun area is inferior to that of CFNet and the proposed method. Overall, CFNet and the proposed method produce better fusion results. However, the proposed method cannot fully recover details in the strongly saturated sun region, indicating that its ability to recover details in regions affected by extreme exposure still requires further improvement.

From the signal intensity analysis, it can be observed that because the GT image exhibits lower brightness in the building surface region, methods such as TransMEF and GIFNet, whose overall pixel intensities are lower than those of the GT, produce images that appear globally darker. HALDeR shows pixel intensities most consistent with the GT; however, the building surface still appears slightly dark in subjective perception, making its performance inferior to CFNet and the proposed method. In contrast, the proposed method and CFNet yield signal distributions closer to the GT in high-intensity regions, while being slightly higher in low-intensity regions, leading to a more natural exposure restoration effect. This further verifies the advantage of the proposed unsupervised fusion strategy, which alleviates the limitation caused by the GT image.

**Figure 10:**
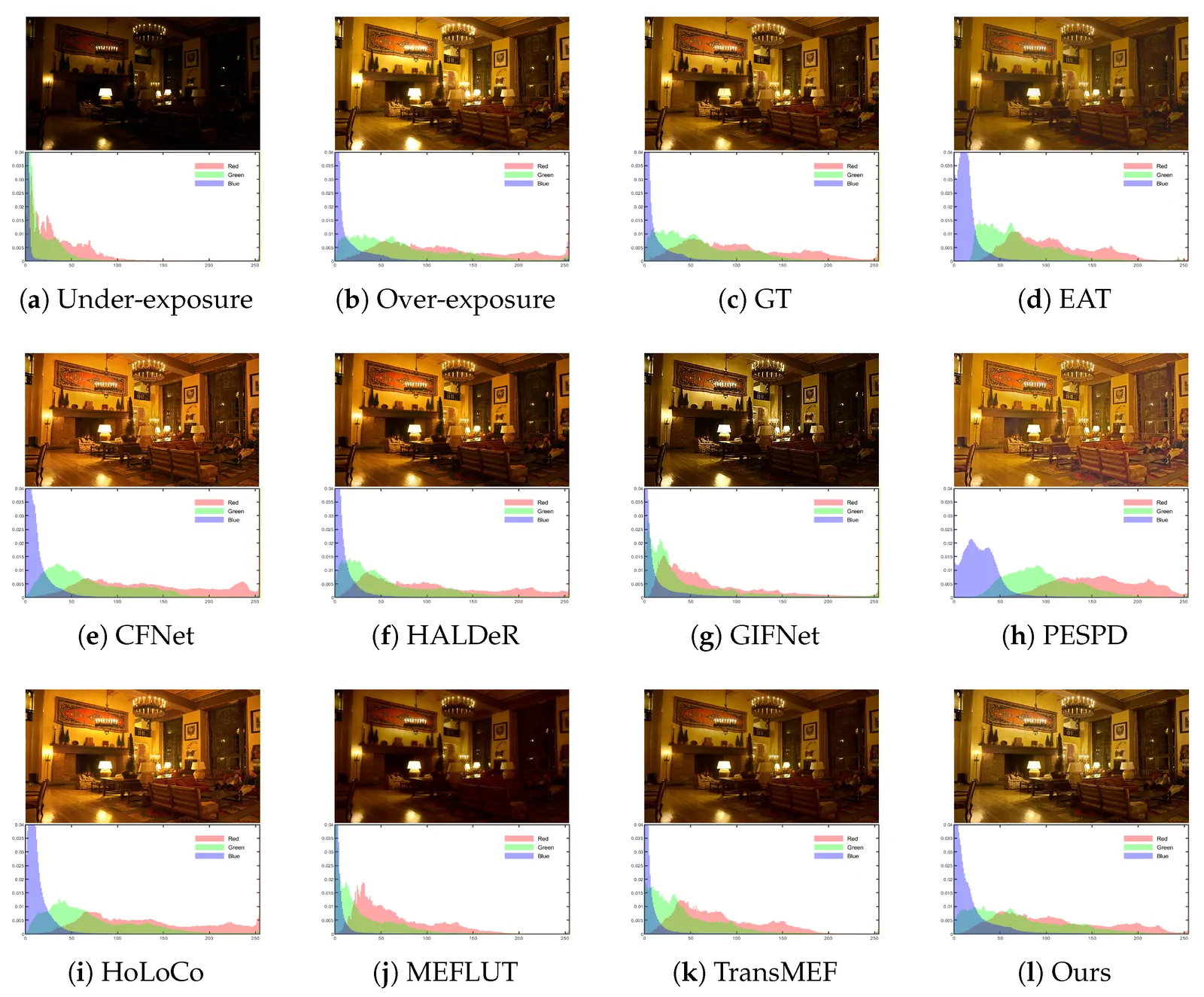
Visual comparison of different MEF methods on the “Living Room” scene. Each result displays the fused image alongside its corresponding RGB three-channel color analysis. The horizontal axis of the graph represents pixel intensity, while the vertical axis denotes the amount of pixels of a given color and intensity as a proportion of all image pixels.

An analysis of the RGB channels of the fusion results was conducted to validate the color preservation performance of the proposed method. As illustrated in Figure 10, the fusion results on the “Living Room” scene produced by GIFNet, MEFLUT, and TransMEF exhibit relatively low pixel intensities, resulting in dark visual appearances and partial loss of image information. Although HALDeR, CFNet, and HoLoCo produce images with satisfactory brightness, they exhibit a distinct red color bias, as confirmed by the significant increase in pixel intensity within the red channel of their color histograms. Overall, our method and EAT produce visually pleasing results, with color histograms that exhibit the closest consistency with the GT.

**Figure 11:**
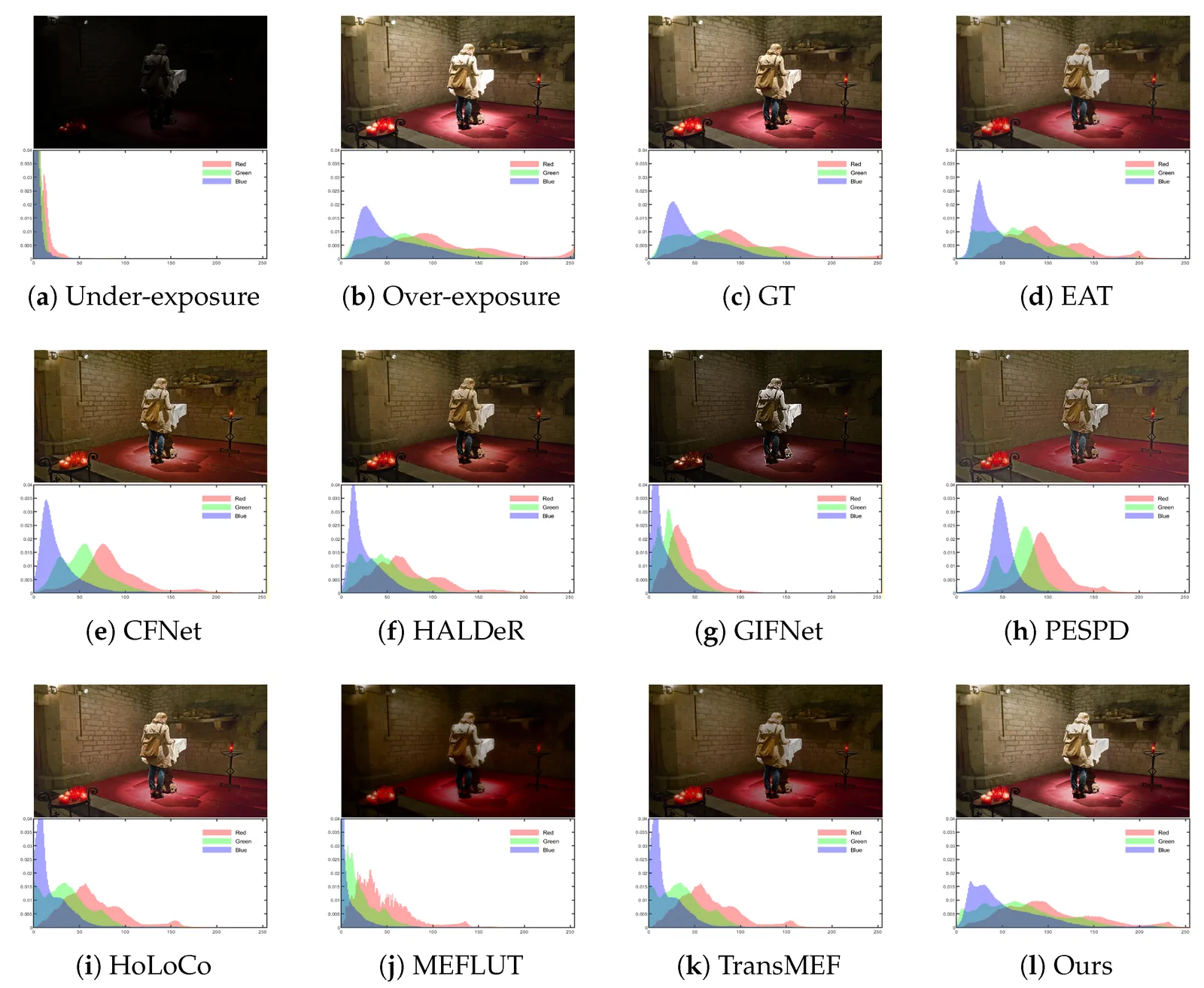
Visual comparison of different MEF methods on the “Chapel” scene. Each result displays the fused image alongside its corresponding RGB three-channel color analysis. The horizontal axis of the graph represents pixel intensity, while the vertical axis denotes the amount of pixels of a given color and intensity as a proportion of all image pixels.

In the results on the “Chapel” scene, shown in Figure 11, it can be observed that due to the under-exposure of the input images, most fusion results appear relatively dark. Among all the methods, our method and HoLoCo achieve the best overall color preservation in the fused images. However, HoLoCo suffers from noticeable image blurring. A comparison of the RGB color analysis histograms further reveals that many of the fusion results exhibit a generally lower pixel intensity distribution, whereas our color histogram shows the closest alignment with the GT.

### 4.5. Objective Experiment

In addition to the subjective experiments, objective experiments are conducted using image-quality metrics to assess the fusion performance of each method. Nine representative indicators are employed to perform a multidimensional analysis of the fusion outcomes, which reflects the overall quality of the fused images with respect to information content, sharpness, and structural consistency. The quantitative results obtained on the SICE dataset are summarized in Table 1.

Table 1 shows that PRMEFNet achieves strong overall performance across the objective metrics. Specifically, it obtains the best results in VIF, $Q^{AB/F}$, MEF-SSIM, and FMI, demonstrating its advantages in visual fidelity, source-image edge transfer, structural consistency, and feature preservation. Although PRMEFNet does not achieve the highest values for all metrics, it maintains competitive performance in the remaining evaluations. In comparison, MobileMEF obtains the highest EN and SD values, indicating its advantages in information entropy and contrast. TransMEF achieves the highest MI value, demonstrating its ability to preserve complementary information from the source images, while GIFNet obtains the highest SF and AG values, reflecting stronger spatial activity and local gradient responses. The relatively lower SF and AG values of PRMEFNet indicate a trade-off between local spatial activity and overall naturalness and structural preservation. By balancing local texture enhancement with smooth transitions between differently exposed regions and suppressing halos and excessive enhancement, PRMEFNet produces more moderate local gradient responses. Overall, under the static, aligned, two-exposure image-fusion conditions evaluated in this study, the proposed method achieves competitive performance in texture preservation, luminance restoration, edge transfer, and structural consistency.

To further evaluate cross-dataset generalization, we directly apply the model trained on the SICE dataset to the dataset released with PESPD. The quantitative results obtained on this dataset are presented in Table 2.

Our method achieves the best performance in VIF, $Q^{AB/F}$, and FMI, while remaining competitive in MI, SF, and MEF-SSIM. This cross-dataset experiment demonstrates that the proposed method can effectively generalize to unseen data under static two-exposure conditions, indicating that the proposed method can learn more generalizable feature extraction and fusion strategies.

### 4.6. Analysis of Fusion Efficiency

To evaluate the fusion efficiency of the proposed method, its fusion speed is compared with that of deep learning-based methods under identical experimental conditions. We selected 100 image sequences from the SICE dataset. Because the original images have relatively high resolutions, both their widths and heights were reduced to one quarter of their original dimensions before testing. After resizing, the image widths range from 750 to 1368 pixels, and the image heights range from 422 to 912 pixels. We calculated the mean inference time and its standard deviation over the 100 sequences to compare the computational efficiency and runtime variability of different methods. The results are presented in Table 3.

The comparison indicates that MEFLUT and the proposed method achieve the fastest average inference time among the evaluated methods. Because both methods use one-dimensional lookup tables for acceleration, they convert the complex model weights into mappings from pixel intensity to fusion weights and perform fusion through one-dimensional lookup. Therefore, the two methods have essentially the same computational cost and the same inference speed. Moreover, the small standard deviations of the two methods indicate that they have more stable inference times and are relatively insensitive to changes in input-image resolution. A more detailed analysis of the parameter count, FLOPs, model memory consumption, and module-level parameter distribution is provided in Appendix A.

### 4.7. Ablation Experiment

To verify the impact of each module in the proposed method on the final fusion performance, a series of ablation experiments are conducted. The complete model is divided into three variants: w/o HFEM, w/o GFEB, and w/o LCFM.

**Figure 12:**
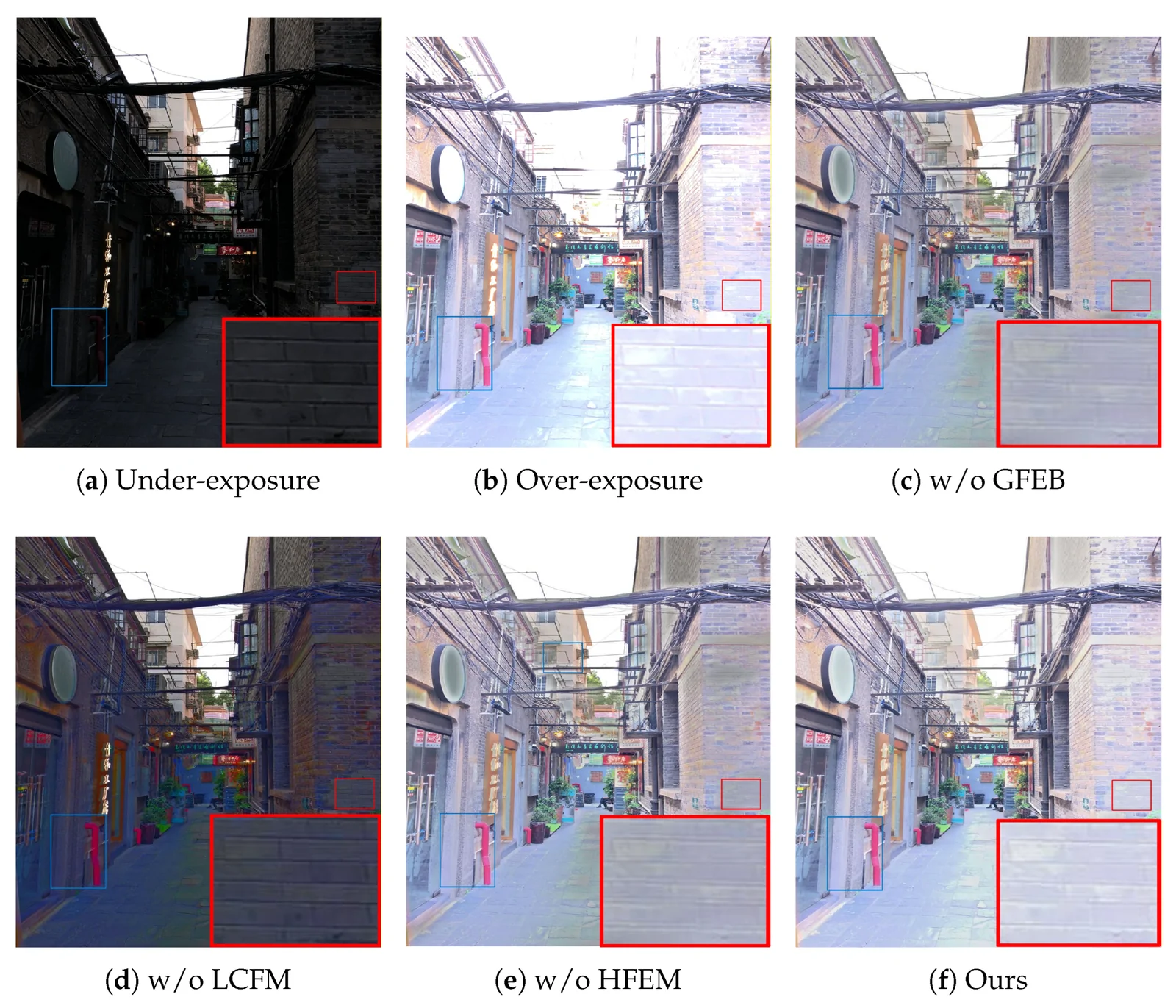
Ablation results on the “Street” sequence.

To intuitively demonstrate the effect of each module on the fusion results, two representative scenes are selected for subjective comparison. The first scene, named “Street”, is used to evaluate the model’s detail recovery capability; while the second scene, named “Grassland”, is selected to assess the model’s ability to suppress halo artifacts. As shown in Figure 12, in the wall region, the fusion result generated by w/o HFEM exhibits obvious texture confusion, with a significant decline in detail recovery. The result of w/o GFEB shows a partial alleviation of texture confusion due to the effect of the HFEM module, but the overall image still appears somewhat blurred. Although w/o LCFM preserves texture details, the absence of contextual feature fusion prevents the model from restoring correct exposure and color consistency, leading to unnatural color shifts and artifacts in the pipe region marked by the blue box. Additionally, its fusion result exhibits extremely uneven brightness in both the sky and street regions.

**Figure 13:**
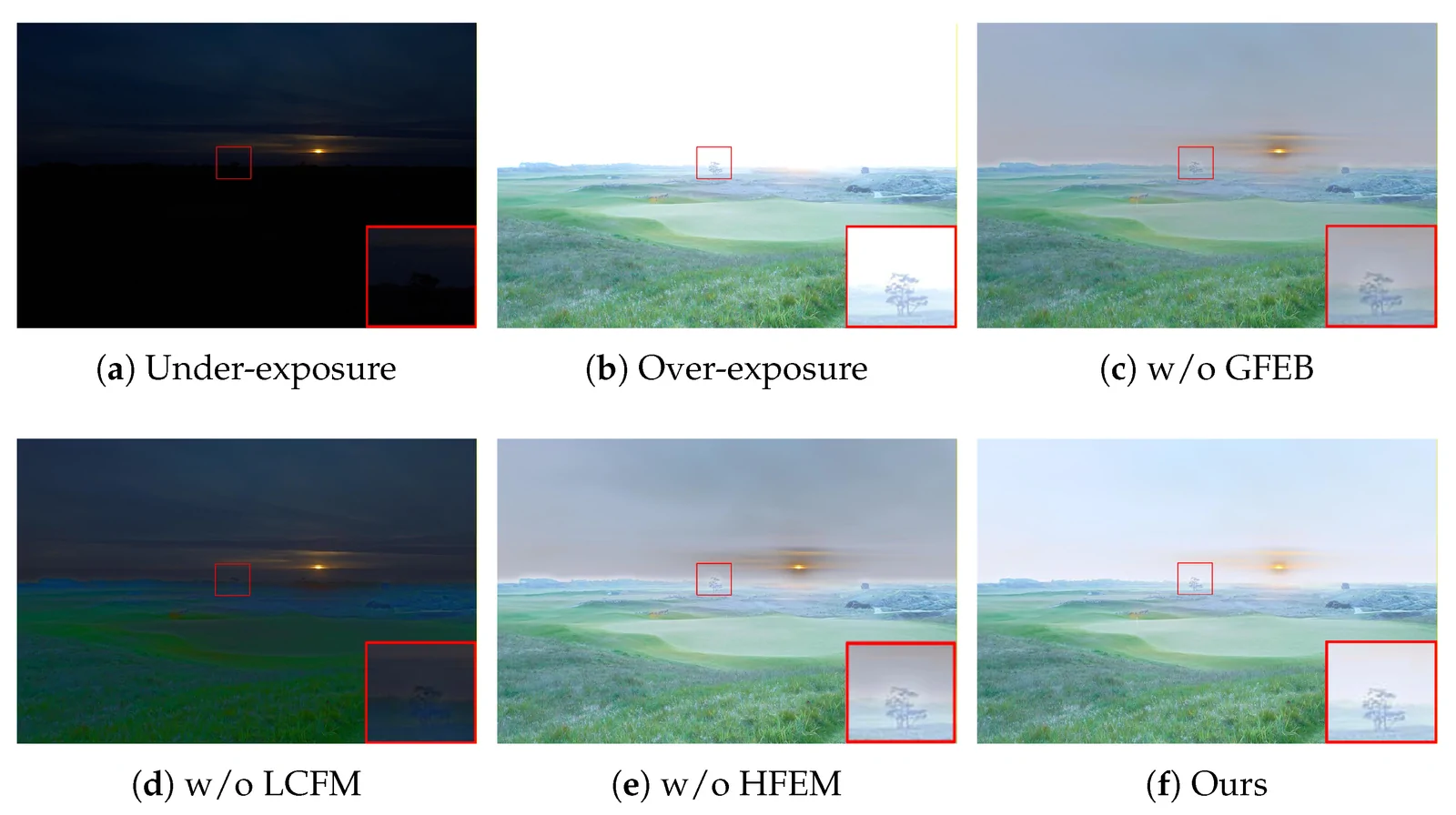
Ablation results on the “Grassland” sequence.

As shown in Figure 13, in the “Grassland” scene, the illumination varies sharply at the boundary between bright and dark regions, demonstrating the model’s capability in suppressing halo artifacts. The results show that the fusion images generated by w/o HFEM and w/o GFEB exhibit noticeable halo artifacts around the horizon and trees, indicating that the proposed model struggles to achieve smooth luminance transitions when either detail extraction or global recovery capabilities are absent. In w/o LCFM, the model lacks contextual modeling capability and fails to restore appropriate luminance. In contrast, the complete model proposed in this study not only avoids large-scale halo artifacts near the horizon but also maintains good performance around small objects, achieving natural light–dark transitions and realistic color reproduction.

To further analyze the role of each module, a quantitative evaluation of the ablation experiment results is conducted, as presented in Table 4.

The complete model yields superior performance on all evaluation metrics. When the HFEM is removed, metrics such as EN, MI, SD, and VIF decrease significantly, reflecting a weakened ability to extract detailed texture information and a reduction in the overall information content of the fused image. After removing the GFEB module, which is composed of dilated convolutions, the global structural consistency of the fusion result declines, with perceptual metrics such as MEF-SSIM and $Q^{AB/F}$ dropping noticeably. This demonstrates that global feature extraction is essential for exposure recovery and structural preservation. In the case of w/o LCFM, all metrics show a substantial decrease, indicating that the feature fusion capability of this module plays a critical role in maintaining model performance. By integrating all three modules, the complete model achieves optimal performance in terms of information content, structural consistency, and perceptual quality, verifying the rationality and necessity of the collaborative design of the submodules.

To further validate the rationale for removing the luminance component of MEF-SSIM from the loss function in the proposed method, we conduct an ablation experiment on the luminance component of MEF-SSIM. The results are presented in Table 5.

The improvements in EN and MI after removing the luminance term indicate that the fused image contains richer information and more complementary content. The improvements in SD, SF, and AG indicate enhanced image contrast, texture variations, and local gradient responses, respectively. The improvements in $Q^{AB/F}$, MEF-SSIM, and FMI indicate that the edges, structures, and feature information in the source images are preserved more effectively. Overall, the improvements across all nine metrics indicate that removing the luminance term enables the loss function to focus more on texture and structure reconstruction and achieves better overall fusion performance with the global exposure guidance provided by the GFEB. Additional ablation experiments on different dilation rates, window-based attention, and complete network and 1D-LUT inference are provided in Appendix B.

## 5. Discussion

The proposed PRMEFNet leverages prior knowledge to enhance the performance of MEF models. Unlike the prevalent method of using two separate network branches to independently extract features from under-exposed and over-exposed images, our method regards the input images as different components with statistically stable properties. By introducing structural constraints into the model, each branch is guided to extract different features, thereby achieving more effective feature extraction and improving upon existing architectures. To evaluate the feature extraction capability of PRMEFNet, its attention maps are visualized. The results show that the DFEB produces sharp boundaries in the transition regions between bright and dark areas, confirming the effectiveness of the proposed design in capturing fine details. The feature maps generated by the GFEB are relatively smooth, and when combined with that from the DFEB, the final feature maps preserve fine details and maintain smooth transitions across regions with different brightness levels, effectively suppressing halo artifacts. These results demonstrate that the proposed method can effectively extract different features in a decoupled manner, verifying the feasibility of feature extraction guided by prior knowledge. We further conduct objective evaluations comparing nine representative methods across nine widely used image-quality metrics. Overall, under the static, aligned, two-exposure image-fusion conditions evaluated in this study, the proposed method achieves competitive performance in texture preservation, luminance restoration, edge transfer, and structural consistency. In addition, subjective evaluations show that our model exhibits strong texture preservation and effective halo suppression, further demonstrating its excellent fusion performance.

This study explores the feasibility of a dual-driven MEF network that integrates prior knowledge with data-driven learning. The results indicate that introducing prior knowledge can effectively guide the model toward enhanced performance, alleviating the reliance of performance enhancement on higher model complexity and providing a new perspective for the design of MEF models.

## 6. Conclusions

This study presents an unsupervised MEF method guided by prior knowledge. By incorporating prior knowledge from traditional methods and leveraging 1D-LUT, the proposed method significantly reduces inference time while maintaining fusion performance. To prevent the model from disproportionately focusing on either texture details or global features, a dual-branch architecture is employed to extract these two types of features separately. In the texture feature extraction branch, hierarchical feature extraction and windowed self-attention are adopted according to the characteristics of texture features, resulting in effective texture feature extraction. In the global feature extraction branch, a designed multi-scale convolution module is employed to guide the model in extracting global features. The final fused image is then generated through the cooperative integration of these two branches, which effectively improves the model’s ability to balance texture and global features. In the inference stage, 1D-LUT is utilized to accelerate the inference process, achieving significant efficiency gains. Comprehensive evaluations on publicly available datasets demonstrate that our method surpasses most existing methods in both fusion quality and inference efficiency, validating the effectiveness of incorporating prior knowledge. A limitation is that the current work does not consider dynamic scenes. Future research will further explore the use of prior knowledge and 1D-LUT in dynamic scenarios.

## Figures and Tables

**Table 1:** Objective experimental results of the proposed and compared methods. The best, second-best, and third-best results are indicated by boldface, underlining, and italics, respectively. The upward arrow indicates that a higher value is better.

Method	EN ↑	MI ↑	SD ↑	SF ↑	VIF ↑	AG ↑	$Q^{AB/F}$↑	MEF-SSIM ↑	FMI ↑
PESPD	6.741	3.993	32.564	*13.637*	1.855	4.440	*0.513*	0.939	0.907
TransMEF	6.618	**6.831**	36.030	8.890	1.660	2.855	0.413	0.927	*0.905*
HALDeR	6.855	6.186	41.371	11.963	*1.809*	3.914	0.526	0.953	0.904
CFNet	6.819	4.488	38.089	13.400	1.712	4.798	0.492	*0.965*	0.884
MEFLUT	6.754	5.681	42.942	7.038	1.435	2.203	0.248	0.866	0.902
EAT	6.628	6.589	36.697	9.900	1.668	3.253	0.488	0.945	0.903
HoLoCo	*6.978*	4.479	42.564	8.980	1.464	3.536	0.331	0.923	0.874
GIFNet	6.702	5.151	*44.388*	**16.935**	1.354	**4.866**	0.329	0.869	0.887
MobileMEF	**7.271**	4.991	**51.512**	9.945	1.736	3.785	0.420	0.970	0.890
Ours	6.988	*6.208*	48.572	14.438	**1.893**	*4.573*	**0.613**	**0.981**	**0.910**

**Table 2:** Objective evaluation results of cross-dataset experiments on the dataset released with PESPD. The best, second-best, and third-best results are indicated by boldface, underlining, and italics, respectively. The upward arrow indicates that a higher value is better.

Method	EN ↑	MI ↑	SD ↑	SF ↑	VIF ↑	AG ↑	$Q^{AB/F}$↑	MEF-SSIM ↑	FMI ↑
PESPD	7.189	3.406	47.061	21.190	*1.230*	**6.880**	0.693	**0.947**	0.891
TransMEF	7.125	**6.439**	56.830	13.616	1.155	4.338	0.532	0.921	*0.892*
HALDeR	*7.262*	5.836	61.239	17.709	1.251	5.624	*0.627*	0.946	0.890
CFNet	7.320	4.201	55.395	16.997	1.076	6.429	0.486	0.934	0.866
MEFLUT	6.943	5.655	*65.065*	11.028	1.030	3.203	0.363	0.842	0.894
EAT	7.167	6.309	54.598	14.747	1.161	4.794	0.611	0.935	0.891
HoLoCo	7.261	4.869	58.251	14.955	1.131	5.226	0.533	0.927	0.881
GIFNet	6.791	5.417	**80.536**	**24.779**	0.979	*6.337*	0.372	0.857	0.881
MobileMEF	**7.457**	4.431	70.112	14.133	1.060	5.268	0.422	0.935	0.879
Ours	7.229	*6.068*	57.337	*18.360*	**1.258**	5.815	**0.695**	*0.941*	**0.902**

**Table 3:** Comparison of fusion times among different methods. Values are reported as mean ± standard deviation. The best and second-best mean values are indicated by boldface and underlining, respectively.

Method	TransMEF	HALDeR	CFNet	MEFLUT	EAT	HoLoCo	GIFNet	MobileMEF	Ours
Time (s)	0.3093 ± 0.0273	0.0981 ± 0.0192	0.4733 ± 0.0353	**0.0018** ± 0.0004	0.0875 ± 0.0852	0.0850 ± 0.0419	2.6721 ± 0.1006	0.1770 ± 0.0111	**0.0018** ± 0.0004

**Table 4:** Objective evaluation of the ablation experiments for each module, where the best and second-best results in each column are indicated by boldface and underlining, respectively. The upward arrow indicates that a higher value is better.

HFEM	GFEB	LCFM	EN ↑	MI ↑	SD ↑	SF ↑	VIF ↑	AG ↑	$Q^{AB/F}$↑	MEF-SSIM ↑	FMI ↑
	✓	✓	6.672	4.889	37.728	14.042	1.773	4.491	0.607	0.964	0.908
✓		✓	6.677	5.082	38.999	13.892	1.789	4.435	0.606	0.966	0.909
✓	✓		5.514	3.743	16.967	6.500	1.303	2.108	0.188	0.834	0.898
✓	✓	✓	**6.988**	**6.208**	**48.572**	**14.438**	**1.893**	**4.573**	**0.613**	**0.981**	**0.910**

**Table 5:** Ablation experiment on the luminance component of the loss function. The best result in each column is indicated by boldface.

Loss Function	EN ↑	MI ↑	SD ↑	SF ↑	VIF ↑	AG ↑	$Q^{AB/F}$↑	MEF-SSIM ↑	FMI ↑
MEF-SSIM	6.640	4.593	34.127	13.300	1.702	4.276	0.591	0.950	0.906
w/o luminance component	**6.988**	**6.208**	**48.572**	**14.438**	**1.893**	**4.573**	**0.613**	**0.981**	**0.910**

## Data Availability

The raw data supporting the conclusions of this article will be made available by the authors on request.

## Appendix A. Computational Complexity Analysis

In this appendix, we provide a detailed analysis of the computational complexity of PRMEFNet. Given that the main text focuses on fusion performance and runtime efficiency, the detailed statistics of the complete network and its functional modules are presented here to support a more comprehensive evaluation of the computational cost of the proposed method.

### Overall and Module-Level Complexity

The complexity measurements are performed using an input tensor of x=[2,1,256,256], corresponding to two single-channel exposure images with a spatial resolution of 256×256. Under this setting, the complete PRMEFNet contains 28.445 M trainable parameters and requires 77.963 GFLOPs for one forward pass. Its parameters occupy 113.779 MB in FP32 format, while the peak GPU memory allocated during a complete forward pass is approximately 221.482 MB. The parameter statistics of the different functional modules are presented in Table A1.

**Table A1:** Parameter statistics of the functional modules in PRMEFNet.

PRMEFNet Component	Params (M)	Proportion of Total Parameters
HFEM	0.005746	0.02%
LCFM	0.182082	0.64%
GFEB	28.256918	99.34%
Complete model	28.444746	100%

As shown in Table A1, HFEM and LCFM together account for only 0.66% of the total parameters, whereas most of the parameters are concentrated in GFEB. Benefiting from the structural constraints imposed by prior knowledge, HFEM and LCFM perform texture-feature extraction and local-context modeling with relatively few parameters. It should be noted that these statistics describe the complete PRMEFNet. During 1D-LUT deployment, the complete network is no longer executed for each input image; instead, the fusion weights are obtained through table lookup and used for weighted fusion. Therefore, the real-time efficiency of the proposed method primarily results from the 1D-LUT deployment.

## Appendix B. Supplementary Ablation Experiments

In this appendix, we provide supplementary ablation experiments on different dilation rates, window-based attention, and 1D-LUT inference. The main text presents the module-level ablation experiments and the ablation experiment on the luminance component of the loss function. To avoid making the main experimental section excessively lengthy, the remaining detailed analyses are presented here to provide a more comprehensive evaluation of the corresponding design choices.

### Appendix B.1. Effect of Different Dilation Rates

To evaluate the influence of different dilation rates in the LCFM, we compare three combinations while keeping all other experimental settings unchanged. The results are presented in Table A2.

**Table A2:** Comparative experiments using different dilation rates in the LCFM. The best result in each column is indicated by boldface. The upward arrow indicates that a higher value is better.

Dilation Rates	EN ↑	MI ↑	SD ↑	SF ↑	VIF ↑	AG ↑	$Q^{AB/F}$↑	MEF-SSIM ↑	FMI ↑
(1,1,1)	6.532	5.252	38.750	13.197	1.795	4.207	0.600	0.975	0.908
(3,6,9)	6.611	5.743	40.543	12.969	1.811	4.132	0.597	0.976	0.902
(2,4,8)	**6.988**	**6.208**	**48.572**	**14.438**	**1.893**	**4.573**	**0.613**	**0.981**	**0.910**

When the dilation rates of all three branches are set to 1, the receptive field of the LCFM is relatively limited, making it difficult to obtain sufficient contextual information. Although the dilation rates of (3,6,9) further enlarge the receptive field, the sparse sampling may omit continuous local structures and thereby affect texture-feature extraction. In contrast, the combination of (2,4,8) achieves a better balance between local sampling density and contextual range and obtains the best results on all nine evaluation metrics.

### Appendix B.2. Effect of Window-Based Attention

To investigate the contribution of window-based attention, we compare the complete model with its variant without window-based attention. All other experimental settings are kept unchanged, and the results are presented in Table A3.

**Table A3:** Ablation experiment on window-based attention. The best result in each column is indicated by boldface. The upward arrow indicates that a higher value is better.

Setting	EN ↑	MI ↑	SD ↑	SF ↑	VIF ↑	AG ↑	$Q^{AB/F}$↑	MEF-SSIM ↑	FMI ↑
w/o window-based attention	6.165	5.283	24.448	8.064	1.521	2.625	0.325	0.898	0.891
Ours	**6.988**	**6.208**	**48.572**	**14.438**	**1.893**	**4.573**	**0.613**	**0.981**	**0.910**

As shown in Table A3, removing window-based attention decreases all nine evaluation metrics. These results indicate that window-based attention enhances the model’s contextual modeling capability, thereby improving its preservation of texture, edge, and structural information.

### Appendix B.3. Complete-Network and 1D-LUT Inference

To evaluate the influence of 1D-LUT deployment on fusion performance, we compare complete-network inference with 1D-LUT inference. The results are presented in Table A4.

**Table A4:** Comparison between complete-network and 1D-LUT inference. The best result in each column is indicated by boldface. The upward arrow indicates that a higher value is better.

Inference Method	EN ↑	MI ↑	SD ↑	SF ↑	VIF ↑	AG ↑	$Q^{AB/F}$↑	MEF-SSIM ↑	FMI ↑
Complete-network inference	6.924	4.580	43.220	14.437	**1.906**	**4.598**	**0.616**	**0.983**	0.908
1D-LUT inference	**6.988**	**6.208**	**48.572**	**14.438**	1.893	4.573	0.613	0.981	**0.910**

The 1D-LUT focuses on establishing a mapping from pixel intensity to fusion weight. This fusion strategy more directly preserves the intensity differences and pixel-level variations in the source image sequence, whereas the complete model uses the extracted abstract features to adjust the fusion weights in specific regions more smoothly. Consequently, the 1D-LUT preserves more pronounced pixel-intensity differences and pixel-level spatial variations from the source images, as reflected by the increases in EN, SD, and SF. Meanwhile, the higher MI and FMI values indicate stronger information dependencies between the fused image and the source images. However, because the 1D-LUT does not include the texture-feature extraction and contextual-modeling processes of the complete network, it cannot refine the fusion weights as precisely according to local edges, gradients, and structural relationships. Therefore, it performs slightly worse than the complete model in VIF, AG, $Q^{AB/F}$, and MEF-SSIM, which are more sensitive to visual fidelity, local gradients, edge transfer, and structural consistency.
